# Maize yield as affected by the interaction of fertilizer nitrogen and phosphorus in the Guinea savanna of Nigeria

**DOI:** 10.1016/j.heliyon.2022.e11587

**Published:** 2022-11-14

**Authors:** Abdullahi Ibrahim Tofa, Alpha Yaya Kamara, Bashir Ahmad Babaji, Kamaluddin Tijjani Aliyu, Temitope Damian Ademulegun, Jenneh Fatima Bebeley

**Affiliations:** a International Institute of Tropical Agriculture (IITA), P.M.B. 5320, Oyo Rd., Ibadan, 200211, Nigeria; b Department of Agronomy, Ahmadu Bello University, P.M.B. 1045, Zaria, 810222, Nigeria; c Centre for Dryland Agriculture, Bayero University, Kano, 700241, Nigeria

**Keywords:** Nitrogen, Phosphorus, N × P interaction, Yield, N use efficiency

## Abstract

The soils of the Nigeria savannas are particularly low in nitrogen (N) and phosphorus (P) and negatively affects maize productivity. The main objective of the study was to evaluate the interactive effect of N and P fertilizers on maize growth, grain yield, nitrogen uptake and N use efficiency. Field experiments were conducted during the 2015 and 2016 cropping seasons at Iburu in southern Guinea and Zaria in northern Guinea savanna zones of Nigeria. The treatments consisted of three levels of nitrogen (0, 60, and 120 kg N ha^−1^) and three levels of phosphorus (0, 13, and 26 kg P ha^−1^). The experimental design consisted of three replications in a split-plot design, with N as the main plot and P as the subplot. Our results show that the response of maize to N depends on the application of P. Higher yields were obtained with the combined application of 120 kg N ha^−1^ and 26 kg P ha^−1^ in both locations. With no P applied, plant N uptake (PNU) was greater at N rate of 120 kg ha^−1^ at Iburu while in Zaria, it increases with increase in N from 0 to 60 kg ha^−1^. When P was applied at 13 kg ha^−1^, the PNU increased by 52 and 66% at Iburu while in Zaria the increases were 51 and 57% each with N application of 60 and 120 kg ha^−1^, respectively, compared with zero N rate. The values for N recovery efficiency (NRE) and agronomic efficiency (AE) were lower for N rate of 120 than for 60 kg ha^−1^ irrespective of P application rate at both locations. The N utilization efficiency (NUTE) however was higher at 120 N kg ha^−1^ under 26 kg P ha^−1^across locations. It can be concluded from these results that in low fertile soils environments such as the Nigeria savannas, N fertilizer should be applied along with P fertilizer for optimum growth, grain yield and nitrogen use efficiency of maize.

## 1 Introduction

Maize (*Zea mays* L.) is an important crop in the Guinea savanna zone of Nigeria but mean grain yield is less than 2 t ha^−1^ due to numerous biotic, abiotic and management constraints [1, 2]. Among several abiotic constraints, inherently low N and available P in the soils have been reported to limit maize yield in the area [3, 4]. Savanna soils are mostly kaolinitic Alfisols with minimal organic matter and cation exchange capacity [5]. The N deficiency has been identified as the major limiting factor for maize productivity in savanna environments [5]. Land usage is becoming more intensive as a result of rising population pressure. The continual usage of the lands has resulted in nutrient and organic matter depletion in the soil, limiting soil productivity and agricultural production. The annual loss of maize yield due to N stress has been reported to range between 10 and 50% [4]. Nitrogen deficiency in Nigeria savannas is caused by a variety of factors, including runoff and leaching of soil N below the root zone as a result of excessive rains [6], poor weed management in farmers' fields [7, 8, 9] and the use of sub-optimal quantities of inorganic fertilizer due to high pricing [10] and non-availability of fertilizer.

After nitrogen, phosphorus is the second most limiting plant nutrient in crop production in most agricultural soils in the Guinea savanna (GS) of Nigeria [11]. A study by Shehu et al. [12] showed very low P levels (less than 3 mg kg^−1^) in most soils in northern Nigeria's savanna. Another study found that P levels were 7 mg kg^−1^ or lower in 93% of study sites in the Sudan savanna and 92% in the northern GS of Nigeria [13]. According to Ekeleme et al. [2] 80% of fields in Nigerian savannas had P levels that were either low or very low.

Phosphorus deficiency has been shown to reduce crop response to applied N by interfering with photosynthetic activity, resulting in reduced growth and yield [14, 15]. According to Fosu-Mensah et al. [16], applying 30 kg P ha^−1^ significantly increased maize response to N application in Ghana's coastal savannas. Mengel et al. [17] reported that when P and K were not applied, the grain yield response to applied N was moderate. According to Onasanya et al. [18] maize crop responds effectively to nitrogen and phosphorus fertilization and thereby resulting in increased yield. They recommended 120 kg N ha^−1^ and 40 kg P ha^−1^ for optimal maize grain output in southern Nigeria.

Fertilizer rate and type [19], crop genetic makeup and physiological components [20, 21], availability of other nutrients [22, 23], soil physical properties [24, 25] and crop management practices [24, 26, 27] can all have major influence on nutrient use efficiency. The application of N increased nitrogen use efficiency (NUE) by 13–96% [28, 29]. However, because of the significant influence of N on maize performance, most studies have largely concentrated on the maize response to N, particularly in the savannas of Nigeria where maize production is high. This has resulted to an increasing emphasis on the use of fertilizer formulations with less P content and more N content for maize production in the savannas.

Optimizing the N and P application rates is necessary to achieve maize yield potential. Both N and P applications have been found to considerably improve maize grain production in Nigerian savannas [30, 31, 32]. Kamara et al. [1] and Oikeh et al. [3] reported maize grain yield increases of >100% for the northern GS of Nigeria with the application of adequate quantities of nitrogen fertilizer but others have reported less response [6, 7]. The reduced emphasis on P may affect maize response to N applications, lowering NUE and maize productivity in Nigerian savannas. There is little information on the combine effect of N and P on maize in the Nigeria savannas. The aim of this study is therefore to evaluate the combined effects of different levels of N and P fertilizers on maize growth, grain yield, nitrogen uptake and nitrogen use efficiency.

## 2 Materials and methods

### 2.1 Description of study site

**Figure 1:**
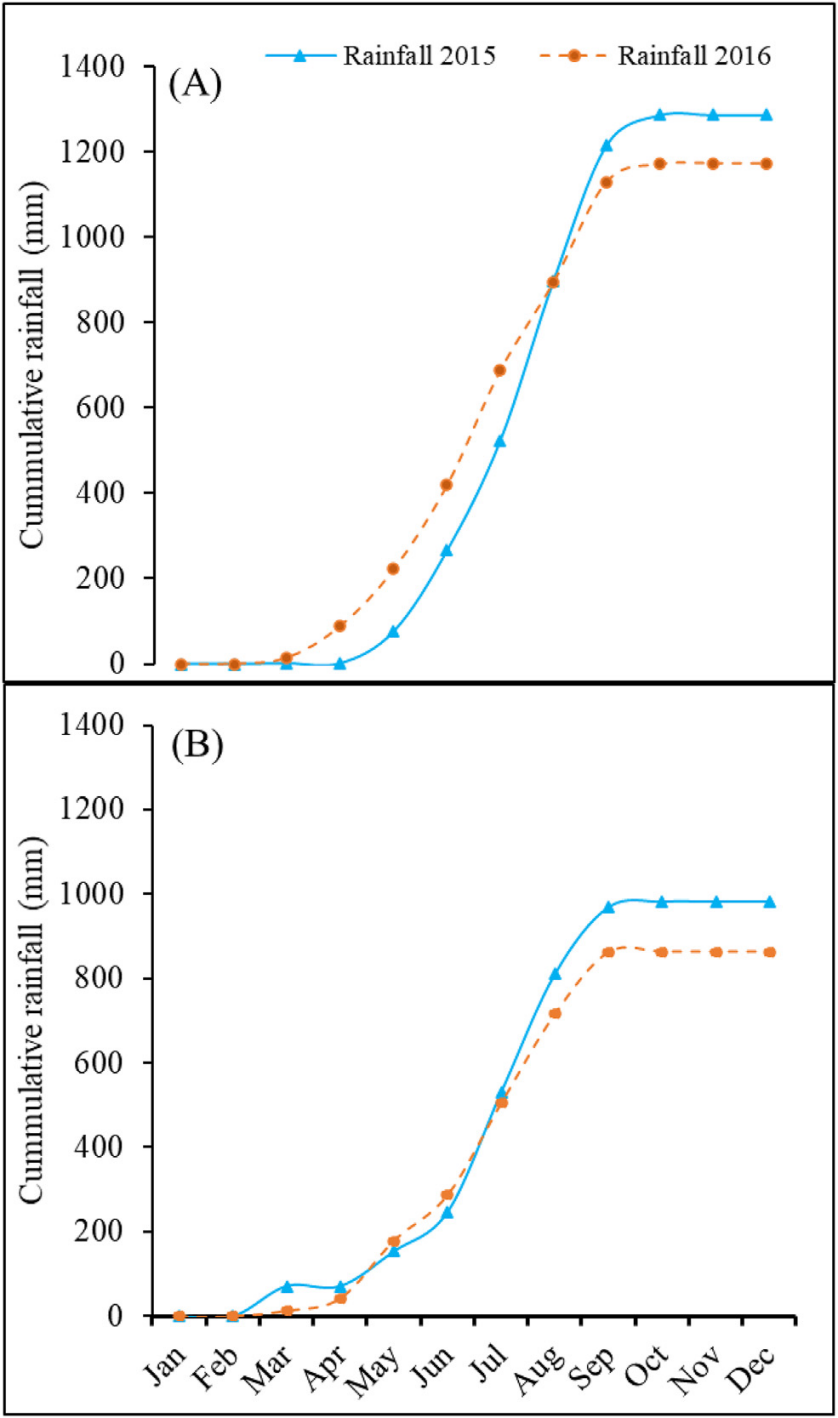
Monthly cumulative rainfall during the two growing seasons (2015 and 2016) at Iburu (A) and Zaria (B), in northern Nigeria. The date of sowing and physiological maturity were June and September, respectively, in both years and locations.

The experiments were conducted during two growing seasons in 2015 and 2016 at Mai Kanti Bello farm Iburu (10° 16′ 12″ N, 7° 47′ 24″ E, 662m a.s.l.) located in the southern GS zone and at Ahmadu Bello University farm, Zaria (11° 3′ 14.4″ N, 7° 42′ 7.2″ E, 686 m a.s.l.) located in the northern GS zone. Within each location, the second-year experiment was established at the same site but adjacent to the previous field. Soil samples were collected at depths ranging from 0 to 30 cm. The soil at Iburu was a lithisol and a loam with 460 g kg^–1^ sand, 370 g kg^–1^ silt, and 170 g kg^–1^ clay and a pH of 4.9. Total soil organic C (OC) was 6.8 g kg^–1^. Total soil N was 0.09 g kg^–1^, the Bray-1 P was 0.54 mg kg^–1^ in 2015. In 2016, the soil test results were similar for the loam soil with 8.0 g kg^–1^ total OC, 0.12 g kg^–1^ total N, and 0.59 mg kg^–1^ Bray-1 P. At Zaria, the soil was lithisol and a loam in both years with 490 g kg^–1^ sand, 330 g kg^–1^ silt, and 180 g kg^–1^ clay with a pH of 5.4, OC = 10.7 g kg^–1^, total N = 0.05 g kg^–1^, and Bray 1 P = 4.47 mg kg^–1^ in 2015. In 2016, the loam soil had pH = 5.3, OC = 10.6 g kg^–1^, total N = 0.11 g kg^–1^, and Bray 1 P = 3.9 mg kg^–1^ (Table 1). Total rainfall at Iburu was 1287 mm in 2015 and 1173 mm in 2016 (Figure 1A). The average minimum and maximum temperatures were 20.3 and 33.2 °C in the experimental periods in 2015 and 20.1 and 33.0 °C in 2016. Zaria received 983 and 863 mm of rain in 2015 and 2016, respectively (Figure 1B). In 2015 and 2016, the average maximum temperature at Zaria was 33.2 °C and 33.0 °C, respectively, and the minimum temperature was 20.3 and 20.1 °C, respectively.

**Table 1:** Physical and chemical analyses of soil at the experimental sites.

	Iburu		Zaria	
Soil properties	2015	2016	2015	2016
Sand (g kg^−1^)	460	360	490	460
Silt (g kg^−1^)	340	480	330	280
Clay (g kg^−1^)	200	160	180	260
Textural class	Loam	Loam	Loam	Loam
pH (H_2_O) 1:2.5	4.90	4.80	5.40	5.28
Organic carbon (g kg^−1^)	6.80	7.95	10.7	10.6
Nitrogen (g kg^−1^)	0.09	0.12	0.05	0.11
Bray-1 P (mg kg^−1^)	0.54	0.59	4.47	3.90
Potassium (K^+^)	0.15	0.11	0.03	0.04
Calcium (cmol^(+)^ kg^−1^)	0.10	0.55	2.34	0.59
Magnesium (cmol^(+)^ kg^−1^)	0.64	0.77	0.64	1.55
ECEC (cmol^(+)^ kg^−1^)	1.42	2.01	3.29	2.72

ECEC = *Effective cation exchange capacity*.

### 2.2 Experimental design and treatments

The experimental design was a complete factorial of three replications in a split-plot design with main plot treatment containing 0, 60, and 120 kg ha^−1^ N supplied as urea (46% N) and 0, 13 and 26 kg ha^−1^ P applied using triple super phosphate (19.89 % P) as the sub-plot treatment. The sub-plot was 3 × 5 m in size, with four rows of 5 m length separated at 0.75 m. The harvesting area measured 1.5 × 4.5 m. Nitrogen was supplied in two halves; half of N rate applied ten (10) days after sowing (DAS). The remaining half was applied 45 DAS. Both phosphorus (single super phosphate) and potassium (muriate of potash) were applied 10 DAS. Potassium was applied at a rate of 50 kg ha^−1^ to all plots. The variety SAMMAZ-15 a medium maturity (106–110 days) was used for the study.

### 2.3 Field management and sowing

The land was harrowed twice and ridged using a tractor before laying out the experimental plots. Before planting, the maize seeds were dressed with Apron Star 42 WS (200 g kg^−1^ Thiamethoxam + 200 g kg^−1^ Metalaxyl–M + 20 g kg^−1^ Difenoconazole, produced by Syngenta Crop protection AG, Basel, Switzerland) at a rate of 2.5 g kg^−1^. In 2015, Sowing was done on June 17 in Iburu and on June 16 in Zaria. During the 2016 cropping season, sowing was performed on June 24 at Iburu and June 18 at Zaria. Two seeds per hole were sown by hand to a depth of about 5 cm and then the seedlings were thinned to one plant per stand at 2 weeks after sowing (WAS). The distance between rows was 0.75 m and the seeding spacing within row was 0.25 m, resulting in a plant density of 53, 333 ha^−1^. To control weeds, a mixture of gramaxone (1:1-dimethyl-4,4-bipyridinum dichloride), and primextra (Atrazine 223 g L^−1^ + Metolachlor 277 g L^−1^) both manufactured by Syngenta Crop protection AG, Basel, Switzerland at a rate of 1 L ha^−1^ each was applied immediately after sowing using a knapsack sprayer. Hand weeding was carried out at 4 and 8 WAS to control the subsequent weeds that emerged in the field. When the incidence of fall armyworm (*Spodoptera frugiperda*) was observed in 2016 cropping season, maize plants were sprayed four times with Ampligo (100 gL^-1^ Chlorantraniliprole +50 g L^−1^ lambda-cyhalothrin, produced by Syngenta) at a spray volume of 300 L ha^−1^.

### 2.4 Agronomic data and measurements

The AccuPAR model LP-80 PAR/LAI Ceptometer (Decagon Devices, Pullman, WA) was used to measure intercepted photosynthetically active radiation (IPAR) and leaf area index (LAI) at the maximum maize tasseling stage. The IPAR was calculated using ceptometer readings of incident light above and under the canopies. Each plot had three measurements collected above and under the canopy, and the observed mean was recorded. Photosynthetically active radiation (PAR) above the maize canopy was taken and averaged from the alleyways with no canopy cover from each plot. For under-canopy PAR, the probe was placed over the two inner rows with the tip of the probe aligned with the plant line of each row. For each plot, the LAI values displayed by the machine were recorded. The ceptometer measurements were taken between 12.00 and 14.00 h, when there were no clouds. The % IPAR in each plot was computed using Eq. (1):

(1) $$IPAR=\left[1.0-\left(\frac{PAR_{b}}{PAR_{a}}\right)\right]\times 100$$

where IPAR is the intercepted PAR, PARa is the PAR (μmol m^−2^ s^−^) measured above maize canopy, PARb is the PAR (μmol m^−2^ s^−1^) measured under maize canopy.

Leaf chlorophyll index was determined using a hand-held chlorophyll meter SPAD (Soil Plant Analysis Development) Model SPAD-502 (Minolta crop, Ramsey, NJ, Illinois U.S.A). SPAD measurement at full tasselling stage was taken from the ear leaves of five randomly selected plants. The reading was taken halfway between the leaf tip and the collar, and halfway between the leaf margin and the leaf midrib [33, 34]. Five measurements were taken from the net plot and averaged to give a SPAD value plant^−1^.

Harvesting was done when the maize kernel reached physiological maturity. During the harvest, a 1.25 m × 1.5 m (1,875 m^2^) quadrat was placed on the two middle rows of the net plot; all plants in a quadrat were harvested to determine grain number m^−2^ and dry matter. Plant samples were divided into cobs, leaves and stems**.** The cobs were shelled, and the seeds, husk, leaves, and stems were oven-dried to a constant weight in a force-draft oven at 60 °C [4] before being weighed with a Mettler Toledo (Model XP60025) balance. The various components were added together to get the total dry matter. The number of grains per unit area of the quadrat in each plot was counted and converted to grains m^−2^. Grain moisture percentage was determined using a portable Farmex MT-16 moisture meter (Farmcomp Oy, Tuusula, Finland). The grain yield of each plot was estimated by adding the grain yield of the quadrat and the grain yield of the remaining net plot. Grain weight was adjusted to 12 g kg^−1^ moisture content. Total dry matter and grain yield were expressed in kg ha^−1^.

### 2.5 Nitrogen use efficiency calculations

The dried subsamples of each plant component (leaves, stems, and grains) were milled using a Thomas Scientific Wiley Mill Model 4 machine. A total C and N analyzer was used to determine the concentration of N in the samples [35]. The total plant N uptake (PNU) was calculated by multiplying the dry weight of the plant parts by the N concentration and summing across parts [1]. For the NUE evaluation, apparent nitrogen recovery efficiency (NRE), agronomic efficiency (AE) and N utilization efficiency (NUTE), were used. The calculations for these parameters were based on Kamara et al. [1], Ciampitti and Vyn [20], and Woli et al. [36]. The treatment outputs were graphed and compared using the Microsoft Excel 2016 software. The NUE parameters were expressed in Eqs. (2), (3), and (4):

Nitrogen recovery efficiency (NRE) is the increase in N uptake in the above-ground plant biomass as relative to fertilizer-N applied (kg kg^−1^) with:

(2) $$NRE\left(kg\;kg^{-1}\right)=\left(\frac{PNU_{N}-PNU_{0}}{N_{rate}}\right)$$

Agronomic efficiency (AE) is the gain in grain yield relative to fertilizer-N applied (kg kg^−1^) with

(3) $$AE\left(kg\;kg^{-1}\right)=\left(\frac{GY_{N}-GY_{0}}{N_{rate}}\right)$$

Nitrogen utilization efficiency (NUTE) is the grain yield produced per unit nitrogen uptake at physiological maturity (kg kg^−1^) with

(4) $$NUTE\left(kg\;kg^{-1}\right)=\left(\frac{GY_{N}-GY_{0}}{PNU_{N}-PNU_{0}}\right)$$

where *PNU*_*N*_ is the total plant nitrogen uptake with N applied, *PNU*_*0*_ is the total plant nitrogen uptake with no N applied, *GY*_*N*_ is the grain yield with nitrogen applied, *GY*_*0*_ is the grain yield with no nitrogen applied and *N*_*rate*_ is the nitrogen rate applied.

### 2.6 Statistical analyses

Using SAS software version 9.3 [37], all data collected was fitted into a general linear model (GLM) and subjected to a combined analysis of variance (ANOVA) across years for each site. Fisher's least significant difference (LSD) test was employed to separate the means when significant F values were found at a significance level of p 0.05.

## 3 Results

### 3.1 Analysis of variance

The ANOVA results in Table 2 show that the Yr × N interaction was significant for total dry matter (TDM), grain yield (GY), total plant nitrogen uptake (PUN), nitrogen recovery efficiency (NER) and agronomic efficiency (AE) at Iburu, and only for grain number per m^2^ and total dry matter (TDM) at Zaria. The Yr × P interaction was significant for SPAD at Zaria and IPAR, LAI and number of grains at both locations. While N × P interaction was significant for LAI, IPAR, number of grains, TDM, GY, and AE at Iburu, at Zaria this interaction was significant for number of grains, GY, PNU and n utilization efficiency (NUTE). Significant 3-way interaction effect was only observed for number of grains at Zaria.

**Table 2:** The ANOVA results for the effects of a fertilizer-N x -P factorial on maize yield conducted for two years at Iburu and Zaria.

Source of variation	SPAD (plant^−1^)	IPAR (%)	LAI (m^2^ m^−2^)	Number of Grains (m^−2^)	TDM (kg ha^−1^)	GY (kg ha^−1^)	PNU (kg ha^−1^)	NRE (kg kg^−1^)	AE (kg kg^−1^)	NUTE (kg ha^−1^)
Iburu										
Nitrogen (N)	∗∗	∗∗	∗∗	∗	∗∗	∗∗	∗∗	ns	∗∗	∗
Yr × N	ns	ns	ns	ns	∗	∗	∗	∗	∗	Ns
Phosphorus (P)	∗∗	∗∗	∗∗	∗∗	∗∗	∗∗	∗∗	∗∗	∗∗	∗
Yr × P	ns	∗	∗	∗	ns	Ns	ns	ns	ns	Ns
N × P	ns	∗∗	∗∗	∗	∗	∗∗	ns	ns	∗	Ns
Yr × N × P	ns	ns	ns	ns	ns	Ns	ns	ns	ns	Ns
Zaria										
Nitrogen (N)	∗∗	∗	∗	∗∗	∗∗	∗∗	∗∗	∗	ns	∗
Yr × N	ns	ns	ns	∗∗	∗	Ns	ns	ns	ns	Ns
Phosphorus (P)	∗∗	∗∗	∗∗	∗∗	∗∗	∗∗	∗∗	∗∗	∗∗	∗
Yr × P	∗∗	∗∗	∗∗	∗∗	ns	Ns	ns	ns	ns	ns
N × P	ns	ns	ns	∗∗	ns	∗∗	∗∗	ns	ns	∗
Yr × N × P	ns	ns	ns	∗	ns	Ns	ns	ns	ns	ns

Yr = year, SPAD = soil plant analysis development, IPAR = intercepted photosynthetically active radiation, LAI = leaf area index, TDM = total dry matter, GY = Grain yield, PNU = total plant nitrogen uptake, AE = agronomic efficiency, NRE = nitrogen recovery efficiency, NUTE = N utilization efficiency.

### 3.2 Physiological parameters

**Figure 2:**
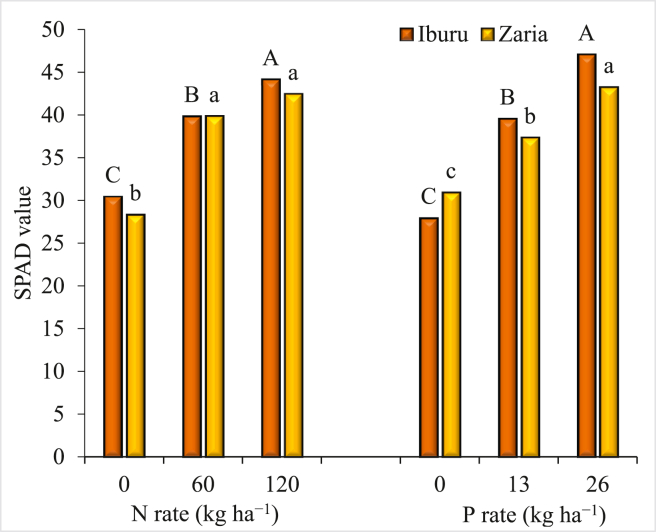
Effect of nitrogen and phosphorus application on soil plant analysis development (SPAD) value at Iburu and Zaria, across two years (2015 and 2016). Within each location different letters indicate a significant difference (p < 0.05) among different treatments using LSD.

The application of N and P significantly increased the SPAD values at both locations (Figure 2). The SPAD values increased with an increase in N rates from 0 to 120 kg ha^−1^ at Iburu; while at Zaria, the values did not considerably increase beyond the N rate of 60 kg ha^−1^. In both study locations, the SPAD values significantly increased with each increase in P from 0 to 26 kg ha^−1^. There was a significant interaction between N and P rates for IPAR and LAI at Iburu (Table 3). When P was not applied, increasing N from 0 to120 kg ha^−1^ did not significantly increase IPAR or LAI. When P was applied at 13 kg ha^−1^, IPAR and LAI significantly increased with application of 120 kg N ha^−1^ compared with 0 kg N ha^−1^. Difference between 60 and 120 kg N ha^−1^ was not significant for IPAR. Similarly, when P was applied at 26 kg ha^−1^, increasing N application from 0 to 60 kg N ha^−1^ significantly increased LAI and IPAR while an increase in N application from 60 to 120 kg ha^−1^ did not result in a substantial change in IPAR and LAI values. The application of N and P at the rates of 120 kg N ha^−1^ + 26 kg P ha^−1^, 120 kg N ha^−1^ + 13 kg P ha^−1^, and 60 kg N ha^−1^ + 13 kg P ha^−1^ produced LAI and IPAR, that were significantly higher than the other treatments (Table 3).

**Table 3:** Interaction effect of nitrogen and phosphorus on photosynthetically active radiation, leaf area index, number of grains, total dry matter and grain yield for maize variety at Iburu, SGS, across two years (2015 and 2016).

	IPAR (%)			LAI (m^2^ m^−2^)			Grains (units m^−2^)			TDM (kg ha^−1^)			Grain yield (kg ha^−1^)		
	P (kg ha^−1^)														
N (kg ha^−1^)	0	13	26	0	13	26	0	13	26	0	13	26	0	13	26
0	41.9	46.1	42.3	0.9	1.1	1.0	574	856	966	2779	3591	3867	491	1218	1615
60	38.2	55.6	66.6	0.8	1.3	1.9	567	1459	1572	3482	7015	8006	955	2685	3259
120	44.6	63.4	73.3	1.0	2.0	2.4	895	1751	1849	5584	8152	9066	1449	3537	4378
LSD_5%_ N × P	17.6			0.6			436.2			1265.2			467.6		

LAI = leaf area index, IPAR = intercepted photosynthetically active radiation, TDM = total dry matter, LSD = Least significant differences.

**Figure 3:**
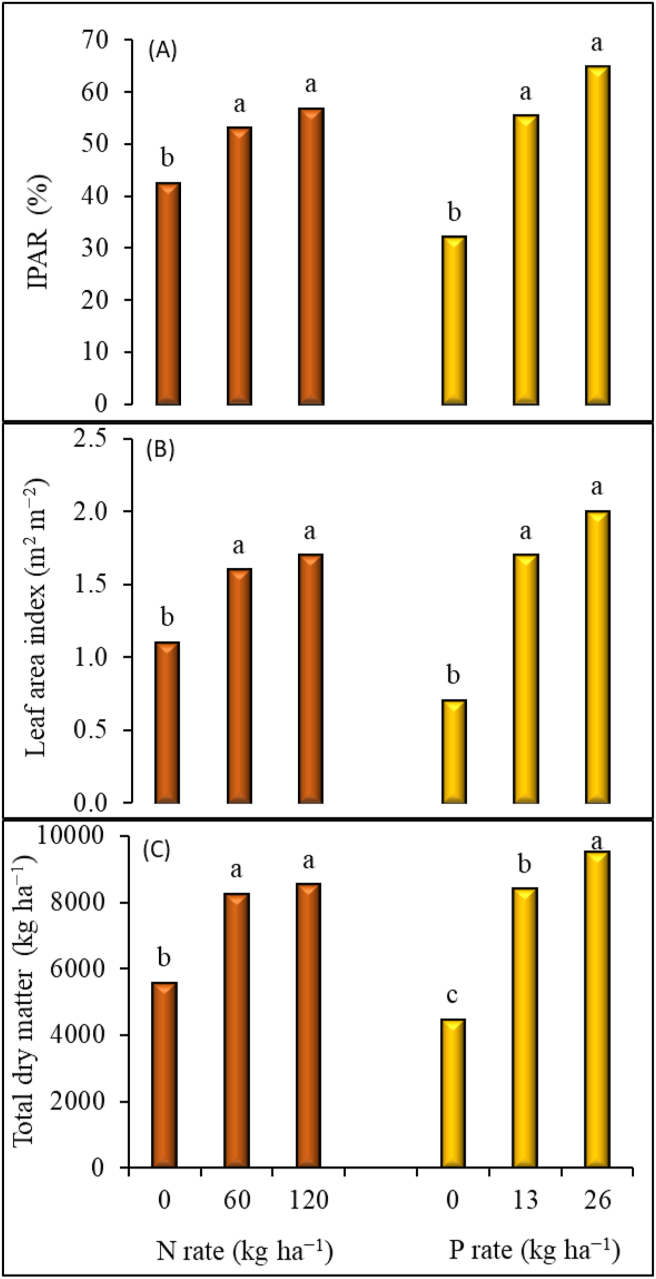
Effect of nitrogen and phosphorus application on (A) IPAR (intercepted photosynthetically active radiation), (B) leaf area index and (C) total dry matter at Zaria across two years (2015 and 2016). Within each treatment different letters indicate a significant difference (p < 0.05) using LSD.

In Zaria, there was no significant interaction between N and P rates for IPAR and LAI. The IPAR and LAI values significantly increased when N rates was increased from 0 to 60 kg ha^−1^, increasing nitrogen from 60 to 120 kg ha^−1^ did not significantly increase IPAR and LAI values. The IPAR increased by 18 and 25% when N was applied at 60 and 120 kg ha^−1^, respectively, compared with zero N rate (Figure 3). Phosphorus application at 13 and 26 kg ha^−1^, respectively, enhanced IPAR by 42 and 50% and LAI by 59 and 65%. In this site, the difference in P rates of 13 and 26 kg ha^−1^ was not statistically significant for both IPAR and LAI.

### 3.3 Grain yield and yield components

The number of grains was significantly affected by the interactive effects of N and P at both locations (Table 2). When no P was applied, differences among N rates were not significant for grains number (Tables 3 and 4). With P application, difference between 0 and 60 kg N ha^−1^ was significant in both location while difference between 60 and 120 kg N ha^−1^ was not significant. The application of P at 13 kg ha^−1^ increased number of grains by 41 and 50% with 60 and 120 kg N ha^−1^, respectively, at Iburu (Table 3). The increase was 56% at both N rates at Zaria (Table 4). The application of P at 26 kg ha^−1^ increased number of grains by 38 and 48% at Iburu, while at Zaria, the increases were 51 and 59% each at N rates of 60 and 120 kg ha^−1^, respectively.

**Table 4:** Interaction effect of nitrogen and phosphorus on number of grains and grain yield of maize at Zaria, across two years (2015 and 2016).

	Grains (units m^−2^)			Grain yield (kg ha^−1^)		
	P (kg ha^−1^)					
N (kg ha^−1^)	0	13	26	0	13	26
0	782	979	1028	663	1684	1743
60	994	2255	2078	1399	3313	3489
120	864	2247	2481	1557	3761	4639
LSD_5%_ N ∗ P	521.6			641.2		

LSD = Least significant differences.

The application of N and P significantly influenced TDM at Iburu (Table 3). When no P was applied, TDM only increased with N application at 120 kg ha^−1^. With application of P at 13 and 26 kg ha^−1^, TDM increased by increasing N rates. However, the application of N beyond 60 kg ha^−1^ did not significantly increase TDM. In Zaria TDM significantly increased with increasing application of N and P but interactive effects were not significant. The TDM increased by 32% with an increase of nitrogen from 0 to 60 kg ha^−1^ and by 35% with increase from 0 to 120 kg N ha^−1^. The Difference between N rates of 60 and 120 kg ha^−1^ was not significant. The TDM significantly increased with each increase in P rate. The TDM increased by 47% when P was applied at 13 kg ha^−1^ and by 60% when applied at 26 kg ha^−1^ compared with 0 kg P ha^−1^ (Figure 3).

The results of the interaction between N and P on grain yield are presented in (Tables 3 and 4). Grain yield response to N was dependent on the application of P in both locations. When no P was applied, grain yield response to N application was significantly higher at N rate of 120 than that of 60 kg ha^−1^ at Iburu (Table 3). In the same location, application of P at both 13 and 26 kg ha^−1^ together with the application of N significantly increased maize grain yield. However, the magnitude of the responses to N were higher at 13 kg than at 26 kg P ha^−1^. The highest yield response was obtained with the application of 13 kg P and 120 kg N ha^−1^. At Zaria, the magnitude of response to N application at 60 kg ha^−1^ was similar among the P rates. The highest grain yield was however obtained with the combined application of 26 kg P and 120 kg N ha^−1^.

### 3.4 Nitrogen use efficiency

**Figure 4:**
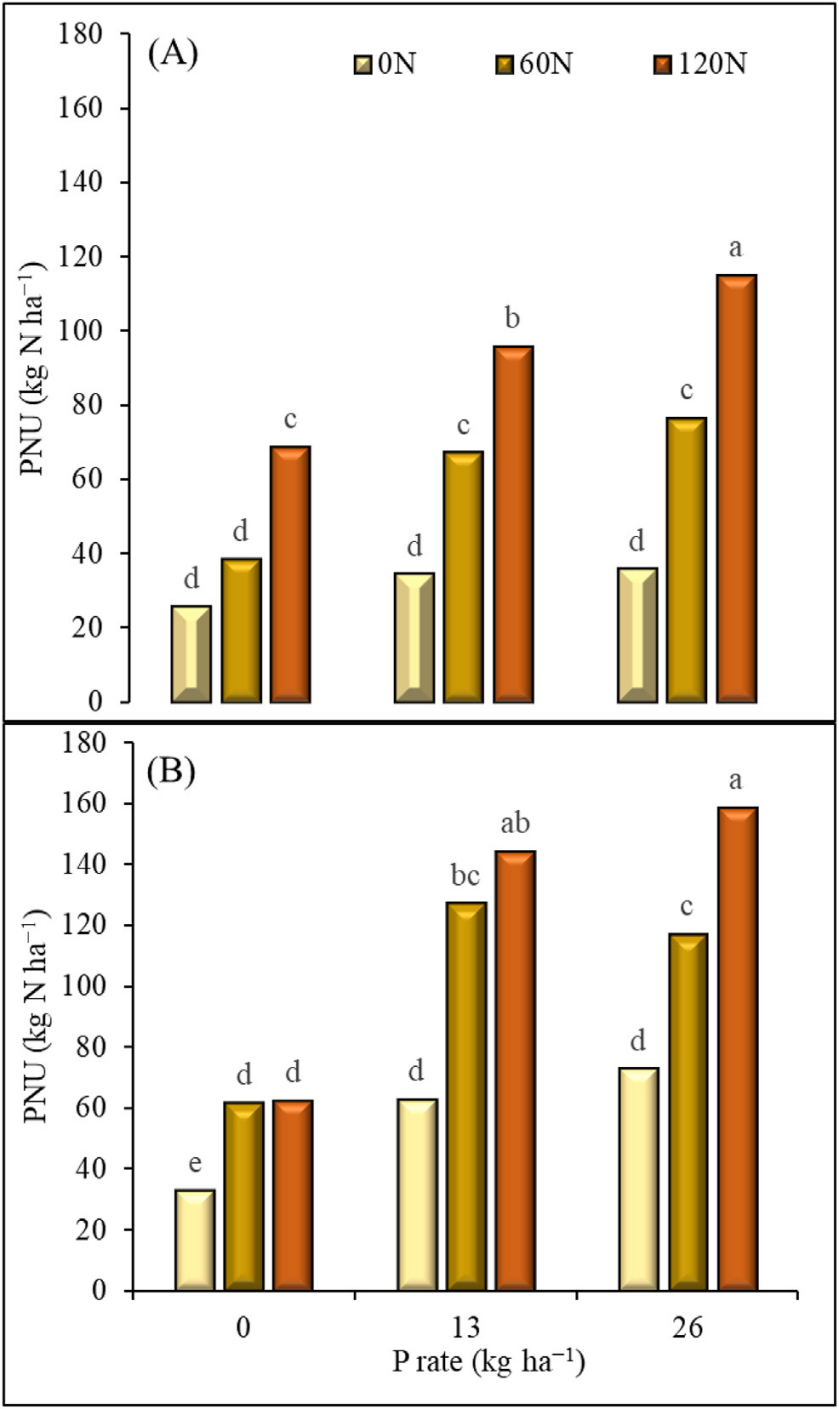
Total plant nitrogen uptake (PNU) for each P and N fertilizer rate at Iburu (A) and Zaria (B), across two years (2015 and 2016). Within each location different letters indicate a significant difference (p < 0.05) among different treatments using LSD.

At both locations, the total plant N uptake (PNU) of maize varied significantly with N and P application rates (Figure 4). At Iburu, PNU was significantly higher at N rate of 120 kg ha^−1^ than at other N rates when no P was applied. The difference in N rates of 0 and 60 kg ha^−1^ was not statistically significant. When P was applied at 13 kg ha^−1^, PNU increased by 43 and 50% for N application rates of 60 and 120 kg, respectively. When P was applied at 26 kg ha^−1^, the PNU increased by 28 and 40% with N application of 60 and 120 kg ha^−1^, respectively (Figure 4A). At Zaria, PNU increased statistically with increase in N from 0 to 60 kg ha^−1^ when no P was applied. The difference between N rates of 60 and 120 kg ha^−1^ was not significant. When P was applied at 13 kg ha^−1^, PNU increased with increase in N rate but difference between 60 to 120 kg N ha^−1^ was not significant. The PNU increased by 51 and 57% for N application rates of 60 and 120 kg, respectively. When P was applied at 26 kg ha^−1^, the PNU increased with each increase in N rate. The increases were 38 and 54% with N application of 60 and 120 kg ha^−1^, respectively, compared with zero N rate (Figure 4B).

**Figure 5:**
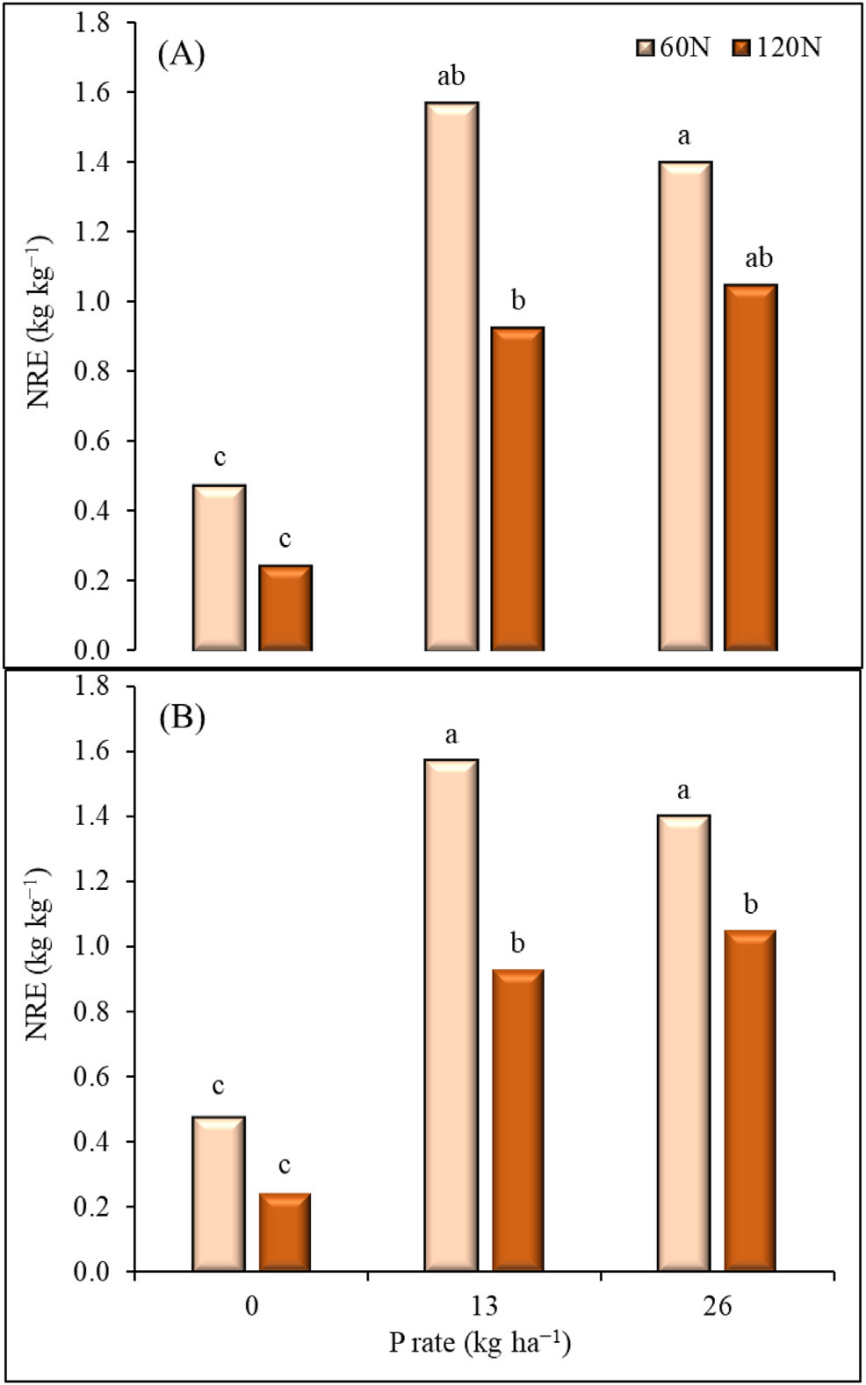
Apparent nitrogen recovery efficiency (NRE) for applied N fertilizer under different P rates at Iburu (A) and Zaria (B), across two years (2015 and 2016). Within each location different letters indicate a significant difference (p < 0.05) among different treatments using LSD.

Nitrogen recovery efficiency (NRE) was significantly influenced by N application at Zaria and by P application at both locations (Figure 5). Lower values for NRE were recorded when no P was applied compared to when P was applied. Differences between N rates of 60 and 120 kg ha^−1^ were not significant when no P was applied. When P was applied at 13 kg ha^−1^, the NRE increased by 70% with application of 60 kg ha^−1^ and 39% with application of 120 kg ha^−1^, at Iburu (Figure 5A). The increases were 69% and 73% with application of 60 and 120 kg ha^−1^, respectively at Zaria (Figure 5B). Phosphorus application of 26 kg ha^−1^ increased NRE by 69% when nitrogen was applied at 60 and by 51% when nitrogen was applied at 120 kg ha^−1^ at Iburu. In Zaria, the increases were 66% and 76%, respectively, when 60 and 120 kg N ha^−1^ were applied.

**Figure 6:**
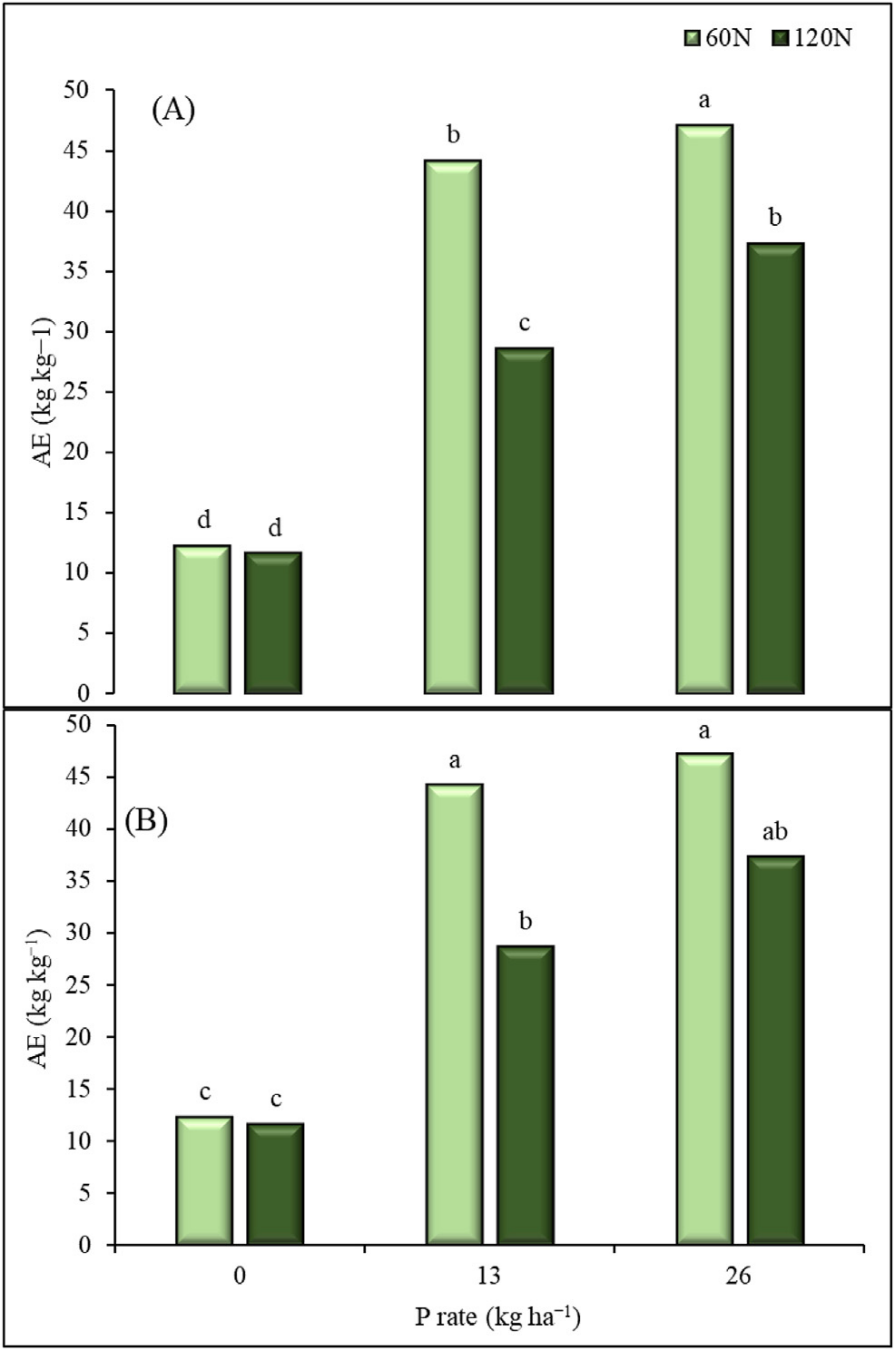
Maize agronomic efficiency (AE) for applied N fertilizer under different P rates at Iburu (A) and Zaria (B), across two years (2015 and 2016). Within each location different letters indicate a significant difference (p < 0.05) among different treatments using LSD.

Agronomic efficiency (AE) was statistically affected by N and P applications. Agronomic efficiency was lower when no P was applied compared with when P application at 13 and 26 kg ha^−1^ (Figure 6). When no P was applied, AE at N application rate 60 kg ha^−1^ did not significantly differ from that of 120 kg ha^−1^. When P was applied at the rate of 13 and 26 kg P ha^−1^, 60 kg N ha^−1^ gave better AE than 120 kg ha^−1^ in both sites. When P was applied at 13 kg ha^−1^, the AE increased by 79% and 69% at Iburu (Figure 6A) when N was applied at 60 and 120 kg ha^−1^, respectively, and by 72% and 59% at Zaria (Figure 6B), for N rates of 60 and 120 kg ha^−1^, respectively, compared to when no P was applied. Agronomic efficiency did not significantly increase when P rate was increased from 13 to 26 kg P ha^−1^, except at Iburu where a combination of 60 kg N ha^−1^ + 26 kg P ha^−1^ gave AE that was significantly higher than that at 60 kg N ha^−1^ + 13 kg P ha^−1^ (Figure 6A).

**Figure 7:**
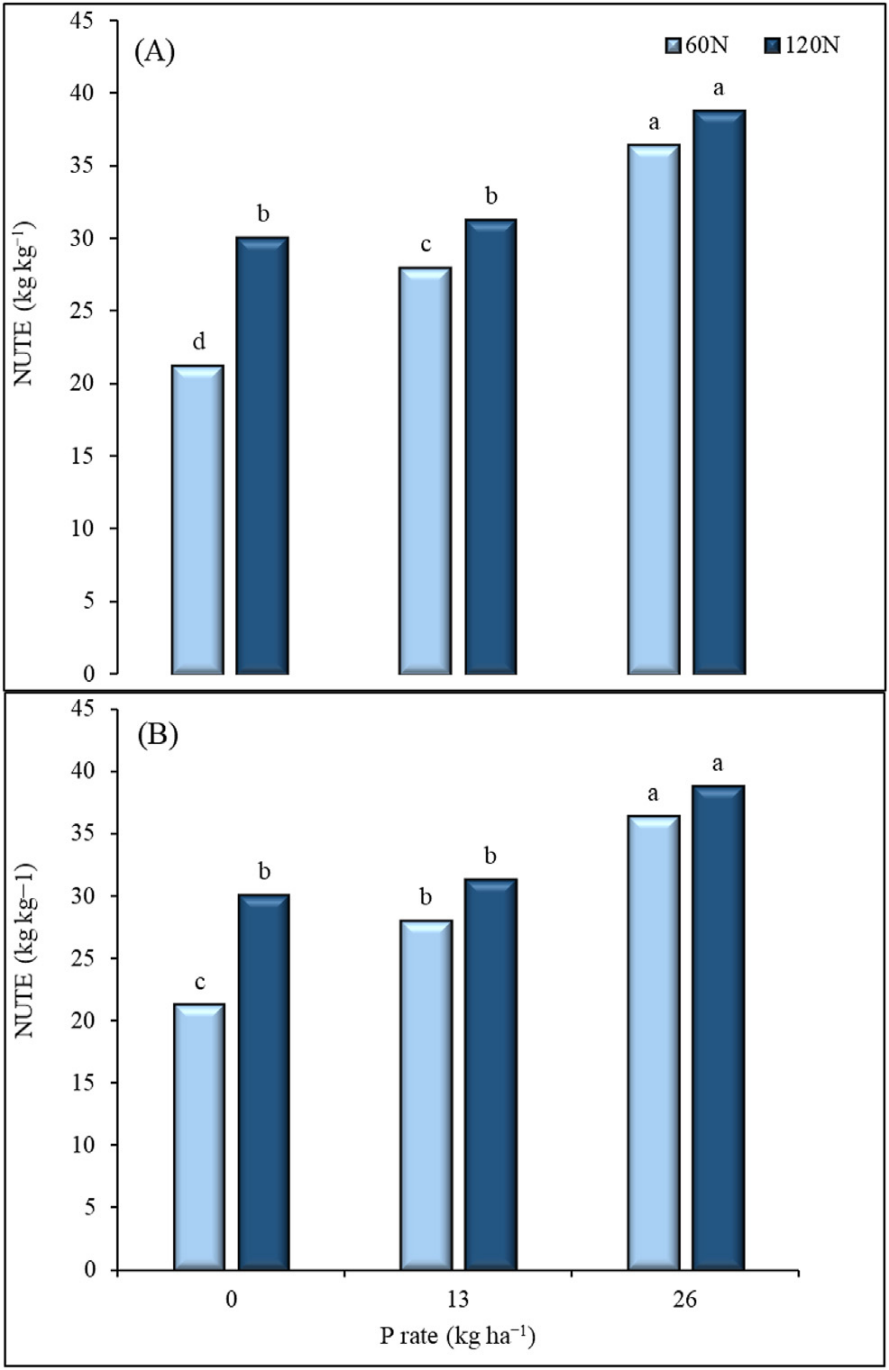
Nitrogen utilization efficiency (NUTE) for applied N fertilizer under different P rates at Iburu (A) and Zaria (B), across two years (2015 and 2016). Within each location different letters indicate a significant difference (p < 0.05) among different treatments using LSD.

NUTE was significantly influenced by both N and P applications across the locations (Figure 7). At P application rate of 0 and 13 kg P ha^−1^, the application of N at 120 kg ha^−1^ did not significantly increase NUTE. Increasing P to 26 kg ha^−1^ resulted to NUTE values that were statistically higher than the other P rates at both locations. At both locations, the difference between 60 and 120 kg N ha^−1^ was not significant at P application rate of 26 kg ha^−1^. At Iburu (Figure 7A), NUTE values for nitrogen rate of 120 kg N ha^−1^ did not significantly differ between P rates of 13 and 26 kg ha^−1^. At Zaria (Figure 7B), NUTE was higher at N rate of 120 kg ha^−1^ than that of 60 kg N ha^−1^. Applying 13 kg P ha^−1^ significantly increased NUTE by 35% with N application of 60 kg ha^−1^ and by 61% with N rate of 120 kg ha^−1^ at Iburu while at Zaria the NUTE increased by 24% at N rate of 60 kg ha^−1^ only, compared with zero P rate. At Iburu, increasing P to 26 kg ha^−1^ did not significantly influence NUTE at both N rates. However, at Zaria, increasing P from 13 to 26 kg P ha^−1^ increased NUTE by 42% and 23% with N rates of 60 and 120 kg ha^−1^, respectively (Figure 7).

## 4 Discussion

Nitrogen and phosphorus are the two most important nutrients that plants need for growth, development and metabolism. Nitrogen is essential to plants nutritional and physiological functions. It also stimulates changes in plant mineral composition and is the most significant factor in growth and development of plants [38]. P application is essential in several plant functions, including energy metabolism, nucleic acid and membrane synthesis, photosynthesis, respiration, nitrogen absorption, and enzyme control [39]. Based on the result of the soil analysis for the two locations, the soils can be generally regarded as low in fertility, particularly in nitrogen and phosphorous which are the focus of this study. This implies that maize response to these nutrients in our study areas could easily be assessed. Lack of significant Yr × N interaction for all the physiological parameters (SPAD, IPAR, LAI) at both locations indicate a consistent response to N irrespective of application rate across the years. The SPAD values differed significantly between all N application rates, with the values increasing with each increase of N application. This shows a progressive response of maize to the N application. This result is comparable to that of Xiong et al. [40] where a very strong correlation between SPAD readings and N leaf contents was reported. Our ANOVA results also show that levels of significance for LAI and PAR over the two locations were the same for all the sources of variation. This is because IPAR depends fully on LAI. In a 3-year study on relationship between IPAR and LAI, Bai et al. [41] reported R^2^ values greater than 0.96 for different cultivars of cotton. The leaf area index and distribution of leaf area within the maize canopy are essential factors that influence total light interception, which effects photosynthesis and production of dry matter [42, 43]. The corresponding higher LAI and IPAR values with increased rates of N and P fertilizers further confirms the complementary effects of the nutrients in maize metabolism which results to observed growth and development. The combined use of N and P helps to maintain functional leaf area and photosynthetic efficiency during the maize growing season [44, 45].

The combination of N and P fertilizers led to a better maize response with respect to grain number and grain yield in the study areas. Across the two sites, the combined application of N (120 kg ha^−1^) and P (26 kg ha^−1^) fertilizers recorded higher values for the number of grains and grain yield. This might have resulted from the higher TDM obtained at higher N and P rates at Iburu and the significant increase in TDM due to increase in N (60 kg ha^−1^) and P (26 kg ha^−1^) at Zaria. The higher grain yield response observed in Iburu at both 13 and 26 kg P ha^−1^ may be attributed to the severe P deficiency in the soil. Result of the soil analysis show a strongly acidic pH level at Iburu that could have favoured the fixation of the P by Al and Fe, thus resulting in the low P [46]. In Zaria, there was no significant difference in yield response between 13 and 26 kg P ha^−1^ with 60 kg N ha^−1^ treatment. However, a significant difference was observed between the 13 and 26 kg P ha^−1^ both under 120 kg N ha^−1^. This may indicate that the 13 kg P was probably sufficient for Zaria. With this it can be understood that yield increase observed between the 13 and 26 kg P ha^−1^ under 120 N could be due to high N application and not P. Aliyu et al. [47] also suggested a P treatment rate of 17 kg P ha^−1^ for maize in similar environment.

The better N and P response obtained at Iburu was mainly because of the lower soil fertility observed in this area. The lower N response obtained in Zaria compared to Iburu when P was applied may be due to the higher clay contents with probably high organic colloidal fractions, which helps retain nutrients in Zaria. According to Kome et al. [48], the physical and chemical characteristics of clay minerals influence soil fertility through regulating nutrient supply and availability [49]. Our results show that the response of maize to N depends on the application of P. Similar to our results, Fosu-Mensah et al. [16] reported that P application significantly increased maize response to N application in coastal savannas of Ghana.

For the nutrient use efficiency indices, Fixen et al. [50] reported a benchmark value of 15–30 kg kg^−1^ for AE. However, higher values than that of the benchmark values were obtained in this study. The higher value of the AE we reported here were obtained at lower N rate of 60 kg ha^−1^. In similar environments to our experiment, Garba et al. [51] reported AE values between 22–38 kg kg^−1^ at 120 kg N ha^−1^ application. These values are also consistent with our result of the AE under the same N application rate. The AE values for N were reported to be high when there is both low and moderate soil N and applied N, respectively [50]. The significant Yr × N for AE at Iburu could be related to yearly variation of rainfall which may leach the nutrient below the maize rooting depth. The AE and NRE values were lower for the 120 kg ha^−1^ N application rate at both 13 and 26 P kg ha^−1^ rates across the locations. However, in relation to PNU, the opposite trend was observed. These outcomes can be related to possible nutrient accumulation. Sattari et al. [52] stated that when supply of a nutrient is higher than for others, the nutrient accumulates in the plant up to a certain mass proportion. Under such situation, the plants' NUTE becomes lower. The established threshold of the NUTE at the maximum accumulation was 35 kg kg^−1^ for the NGS of Nigeria [46]. These values are similar to those we observed in this current study; thus, it can be deduced from the results that the 120 kg N ha^−1^ applied is possibly higher than what is required for the maize to reach its’ maximum NUTE of 79 kg kg^−1^ as similarly contained in Shehu et al. [46]. The lower NRE values for 120 kg N ha^−1^ across locations and irrespective of P rate, further implies N is loss more with the higher application rate. Thus, N management strategy has to be further improved probably by adopting three split applications instead of two splits used in this study. This may assist in avoiding the potential losses of the N through leaching and runoff. This study further showed a general increase in NRE, AE and NUTE when P was applied. Lower values for NUE parameters were recorded when P fertilizer was not applied at all N rates in both sites. This shows the importance of phosphorus fertilizer in the efficient utilization of nitrogen by maize in the savannas of Nigeria. In this study, a combination of 60 kg N and 13 kg P ha^−1^ was found to increase most of the NUE measures. Our results agree with the results of Wen et al. [53], who reported higher N uptake and utilization by maize when optimum N and P were supplied together than when N fertilizer was applied alone.

## 5 Conclusions

There was significant interactive effects of N and P on physiological, nutrient use efficiency and yield response of maize at Iburu and Zaria in the GS zone of Nigeria. Generally, higher yields were obtained with the combined application of 120 kg N ha^−1^ and 26 kg P ha^−1^. This highlights the importance of the two nutrients in the study areas. However, higher values for NRE and AE were recorded at the intermediate application rates of N (60 kg ha^−1^) and P (13 kg ha^−1^). Lower NRE values were recorded for N rate of 120 kg N ha^−1^ across locations and irrespective of P rate. At both locations, the NUTE values did not differ statistically between N rates of 60 and 120 kg ha^−1^ when P was applied at 26 kg ha^−1^. Based on these findings, it can be concluded that a clear response of maize to the applied nutrients was observed. The lower nutrient use efficiencies of N observed at the highest N application rate indicates a potential loss and/or accumulation of N in the plant.

## Declarations

### Author contribution statement

Abdullahi Ibrahim Tofa, Alpha Yaya Kamara, Bashir Ahmad Babaji: Conceived and designed the experiments; Analyzed and interpreted the data; Performed the experiments; Wrote the paper.

Temitope Damian Ademulegun, Jenneh Fatima Bebeley: Performed the experiments.

Kamaluddin Tijjani Aliyu: Analyzed and interpreted the data.

### Funding statement

This work was supported by Bill and Melinda Gates Foundation [OPP1113374] through IITA as part of the Project Taking Maize Agronomy.

### Data availability statement

Data will be made available on request.

### Declaration of interest’s statement

The authors declare no conflict of interest.

### Additional information

No additional information is available for this paper.
